# Assessing asymmetrical kidney function in living donors: a retrospective cohort study on CT metrics

**DOI:** 10.1186/s12882-024-03634-7

**Published:** 2024-07-02

**Authors:** Joseph Sturman, Anthony Fenton, Usman Hayat, Robert Jones, Graham Lipkin

**Affiliations:** 1 Department of Renal Medicine, University Hospitals Birmingham NHS Foundation Trust, Queen Elizabeth Hospital Birmingham, Mindelsohn Way, Edgbaston, Birmingham, B15 2GW UK; 2 Institute of Immunology and Immunotherapy, College of Medical and Dental Sciences, University of Birmingham, Edgbaston, Birmingham, B15 2TT UK; 3 Kidney Unit, Royal Stoke University Hospital, Newcastle Road, Stoke-on-Trent, ST4 6QG UK; 4 Department of Interventional Radiology, University Hospitals Birmingham NHS Foundation Trust, Queen Elizabeth Hospital Birmingham, Mindelsohn Way, Edgbaston, Birmingham, B15 2GW UK

**Keywords:** Kidney transplantation, Living donor, Kidney size, DMSA

## Abstract

**Background:**

Live donor kidney transplantation is the preferred kidney replacement therapy for eligible patients but requires thorough donor evaluation to minimise risks. Contemporary guidelines recommend split kidney function measurement in living donors only when there is a significant kidney size discrepancy, yet the evidence for this is poor, and practice varies nationally. This study evaluates the efficacy of CT-derived kidney metrics in detecting significant functional asymmetry.

**Methods:**

We conducted a retrospective cohort analysis of 123 prospective living kidney donors at a regional transplant centre from June 2011 to October 2014, utilising CT to determine kidney and cortical volumes and lengths. Asymmetric kidney function (AKF), defined by > 10% function difference on DMSA scans, was correlated with CT measurements to calculate the diagnostic accuracy of current guidelines.

**Results:**

Among the prospective donors, the median age was 42 years, and 59.3% were female. The median split kidney function difference was 4%, with 25 individuals exhibiting > 10% AKF. Kidney length discrepancy proved to be a poor indicator of AKF (sensitivity: 28%, specificity: 84%). While negative predictive values for cortical and kidney volumes were high (96% and 93%, respectively), sensitivity was low, and specificity and positive predictive value did not meet satisfactory thresholds.

**Conclusions:**

CT-derived metrics of kidney length, cortical, and total volume show limited sensitivity and specificity in identifying significant AKF. These findings provide evidence to support revised guideline development in the assessment of living kidney donors.

**Supplementary Information:**

The online version contains supplementary material available at 10.1186/s12882-024-03634-7.

## Background

Kidney transplantation is a transformative treatment for patients with end-stage kidney disease (ESKD), offering significantly better quality of life and healthcare outcomes compared to dialysis. Optimal results are often achieved through pre-emptive or early post-dialysis transplantation enabled through a carefully assessed and counselled live donor [1]. Accurate assessment of donor kidney function is critical to minimise the risk to the donor, and guidelines have been published by ‘Kidney Disease: Improving Global Outcomes’ (KDIGO) and the UK British Transplantation Society [2, 3]. These guidelines suggest that differential kidney function, evaluated via technetium-99m 2,3 dimercaptosuccinic acid (^99m^Tc-DMSA) scans, should be specifically conducted when there is more than a 10% size variation or when significant anatomical abnormalities are detected. However, such split kidney function measurement is not uniformly mandated, and the underpinning evidence for these recommendations remains substandard. Moreover, the correlation between kidney size from pre-operative imaging and actual differential kidney function is not well-established, leading to inconsistent evaluation practices across transplant centres [4–6].

In clinical decision-making, the usefulness of a test is dependent upon its sensitivity, specificity and positive/ negative predictive values which give vital information about the degree of certainty a positive or negative finding confers. Understanding this data is therefore pivotal in future guideline development regarding the use of computed tomography (CT)-derived measurements as a proxy for split renal function. Addressing this gap, our study examines the effectiveness of CT-derived kidney length and volumes in detecting differential kidney function in prospective living donors, aiming to bolster the evidence base for future guideline refinement.

## Methods

In this retrospective analysis, we examined a consecutive series of individuals assessed for living kidney donation at the University Hospitals Birmingham, Queen Elizabeth Hospital, UK, from June 2011 to October 2014. Potential donors were identified through a comprehensive review of electronic radiology and clinical records. Standard pre-donation evaluations included ^99^^m^Tc-DMSA scans for renal cortical scintigraphy and measured glomerular filtration rate (mGFR) assessments via chromium-51 labelled ethylenediamine tetraacetic acid (^51^Cr-EDTA) plasma clearance, in line with established protocols.

All participants also received arterial phase CT renal angiography and delayed CT intravenous urography, which were conducted on the same day following the nuclear medicine studies. We rigorously cross-checked radiological reports against clinical records to verify the prospective donor status, excluding cases where CT imaging was conducted for other diagnostic reasons. Furthermore, we systematically collated baseline demographic and clinical data from the medical records for analysis.

A quantitative assessment of renal anatomy was conducted using CT imagery to measure kidney length, volume, and cortical volume. Kidney lengths were measured by a single operator, trained and blinded to DMSA results, to mitigate potential bias. This operator adhered to a strict protocol of aligning the kidney axis in the coronal plane for optimal length measurement in the sagittal plane. We utilised the Siemens Leonardo workstation to assess kidney and cortical volumes, which features tissue volume estimation capabilities. This semi-automated process included meticulous delineation of the kidneys’ outer margins, with exclusions for major vessels, collecting systems, and cystic lesions. Cortical volume was derived by subtracting the medullary volume with a different density, allowing Hounsfield unit-based differentiation.

Our criteria for defining asymmetrical kidney bipolar length and volume discrepancies were as per current guidelines [2, 3]. A differential greater than 2 cm or 10% between kidneys was flagged as significant, with the smaller kidney as the reference point to enhance sensitivity. The 10% disparity was also applied to cortical volume, supported by recent literature suggesting this as an abnormal range across various age groups [7].

Finally, DMSA scans, conducted as per British Nuclear Medicine Society standards, served as the benchmark for determining split kidney function [8]. The diagnosis of Asymmetrical Kidney Function (AKF) was based on a > 10% variance in function between kidneys, with the subsequent calculation of diagnostic accuracy metrics for each CT-measured parameter.

## Results

In our cohort of 123 evaluated prospective living kidney donors, 25 (20.3%) exhibited Asymmetrical Kidney Function (AKF). The median age was 42, with the interquartile range (IQR) between 32 and 53, and females comprised 59.3% of the group.

**Fig. 1:**
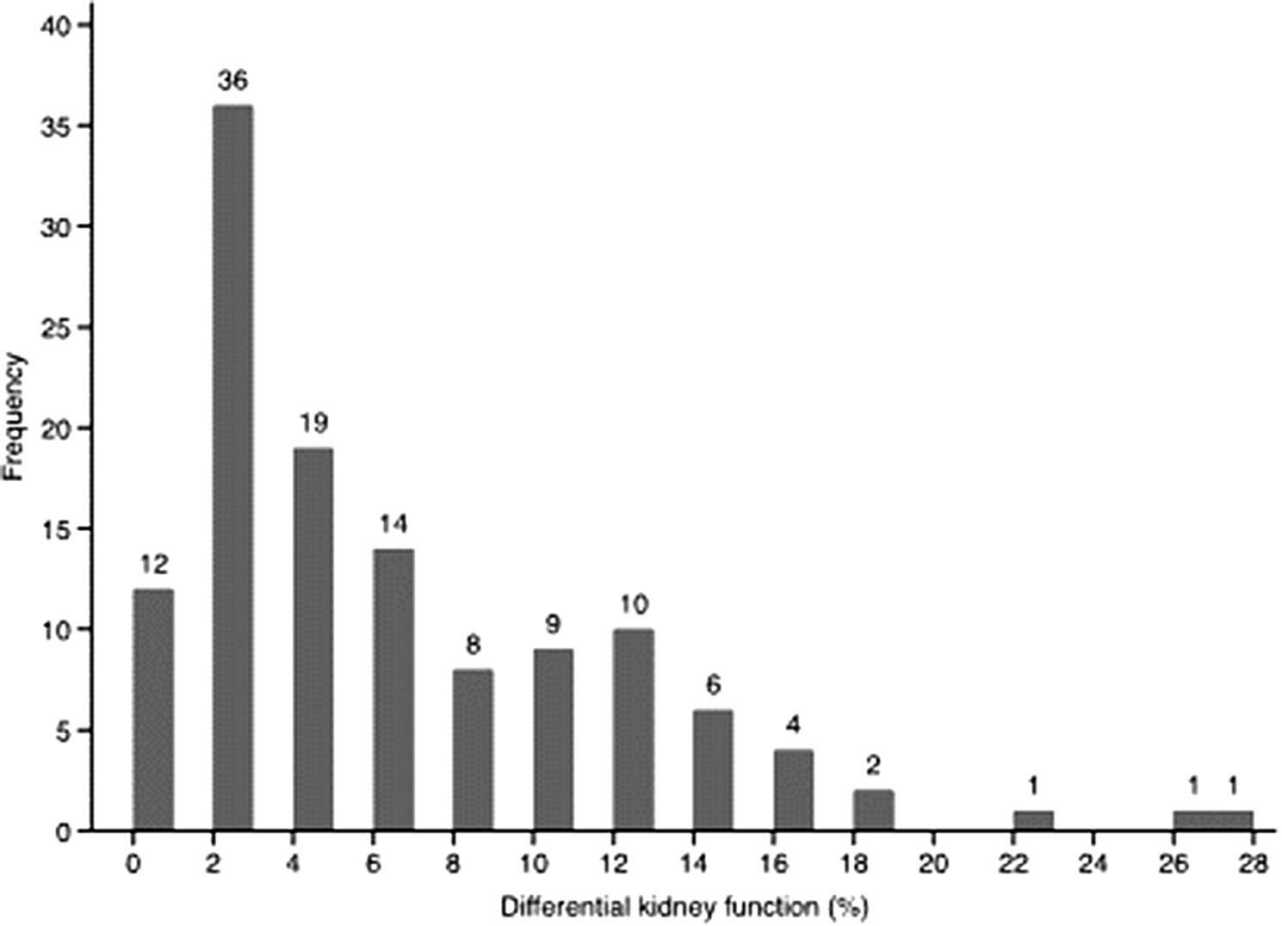
Histogram showing the distribution of differential kidney function in 123 prospective live kidney donors (%)

The differential in kidney function had a median of 4%, ranging from 0 to 28%, and the distribution is depicted in Fig. 1.

No patients had an absolute kidney size difference exceeding 2 cm. For those with AKF, the mean differential split function was 15.2%, with a standard deviation of 4.41.

**Fig. 2:**
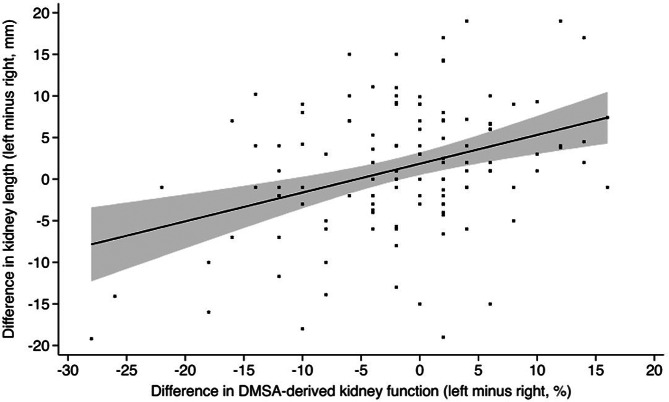
Scatter plot of the difference in bipolar kidney length and DMSA-derived differential kidney function. (Regression line and 95% mean confidence interval in shaded area)

Our analysis also demonstrated a weak positive correlation between bipolar kidney length and differential function (Pearson’s correlation coefficient *r* = 0.363, *P* < 0.001), as visualised in Fig. 2.

In assessing diagnostic accuracy, the sensitivity of a > 10% size difference as a marker for renal function discrepancy was found to be suboptimal at 28% (95% CI: 12 − 49%). Specificity was better but still insufficient at 84% (95% CI: 75 − 90%).

Tables 1, 2, 3, 4 and 5 detail the data on kidney dimensions and the diagnostic performance of the measurements. The contingency tables and diagnostic estimates elucidate the limitations of size-based metrics in predicting AKF, with cortical and total kidney volumes yielding higher sensitivities than bipolar kidney lengths but with limitations in specificity and positive predictive values.

**Table 1:** Cross-tabulation of > 2 cm difference in kidney lengths by Asymmetric Kidney Function (defined as > 10% difference in kidney function)

> 2 cm difference in kidney lengths	Asymmetric kidney function		Total
	Positive	Negative	
**Positive**	0	0	0
**Negative**	25	98	123
**Total**	25	98	123

**Table 2:** Cross-tabulation of > 10% difference in kidney lengths by Asymmetric Kidney Function (defined as > 10% difference in kidney function)

> 10% difference in kidney lengths	Asymmetric kidney function		Total
	Positive	Negative	
**Positive**	7	16	23
**Negative**	18	82	100
**Total**	25	98	123

**Table 3:** Cross-tabulation of > 10% difference in kidney volumes by Asymmetric Kidney Function (defined as > 10% difference in kidney function)

> 10% difference in kidney volumes	Asymmetric kidney function		Total
	Positive	Negative	
**Positive**	20	30	50
**Negative**	5	68	73
**Total**	25	98	123

**Table 4:** Cross-tabulation of > 10% difference in cortical volumes by Asymmetric Kidney Function (defined as > 10% difference in kidney function)

> 10% difference in cortical volumes	Asymmetric kidney function		Total
	Positive	Negative	
**Positive**	22	32	54
**Negative**	3	66	69
**Total**	25	98	123

**Table 5:** Estimates of detection of significant differential kidney function with 95% confidence intervals

Statistic	> 2 cm difference in bipolar kidney lengths		> 10% difference in bipolar kidney lengths		> 10% difference in renal cortical volumes		> 10% difference in total kidney volumes	
	Value	95% CI	Value	95% CI	Value	95% CI	Value	95% CI
**Sensitivity**	**0.00**	0.00 to 0.14	**0.28**	0.12 to 0.49	**0.88**	0.69 to 0.97	**0.80**	0.59 to 0.93
**Specificity**	**1.00**	0.96 to 1.00	**0.84**	0.75 to 0.90	**0.67**	0.57 to 0.76	**0.69**	0.59 to 0.78
**Positive likelihood ratio**	n/a		**1.72**	0.79 to 3.71	**2.69**	1.96 to 3.71	**2.61**	0.18 to 3.73
**Negative likelihood ratio**	**1.00**	1.00 to 1.00	**0.86**	0.66 to 1.12	**0.18**	0.06 to 0.52	**0.29**	0.13 to 0.64
**Positive predictive value**	n/a		**0.30**	0.17 to 0.49	**0.41**	0.33 to 0.49	**0.40**	0.32 to 0.49
**Negative predictive value**	**0.80**	0.80 to 0.80	**0.82**	0.78 to 0.86	**0.96**	0.88 to 0.98	**0.93**	0.86 to 0.97

## Discussion

This study embarked on a critical evaluation of CT-derived kidney length and volume measurements in the context of identifying asymmetric kidney function (AKF) among prospective living kidney donors, a group for whom accurate and non-invasive screening methods are paramount. Despite the potential of CT metrics to streamline donor evaluation by negating the need for radionuclide imaging, they must be highly sensitive to avoid false negative results and potential harm to donors [9, 10]. . Unfortunately, our findings cast doubt on their reliability and predictive value. The observed sensitivity and specificity of these measurements suggest a significant shortfall with existing guidelines recommending differential function assessment primarily based on anatomical discrepancies. The assumption that CT-derived measurements alone can suffice for donor screening, which underlies current practice and guidelines in screening living kidney donors, merits reconsideration.

### Bipolar kidney lengths

Our results demonstrate an apparent inadequacy in the value of kidney length discrepancy for detecting AKF in living kidney donor assessments. The alarmingly low sensitivities (0% for > 2 cm and only 28% for > 10% differences), alongside prohibitively high false negative rates, sharply question the reliability of such measurements. Indeed, with a 100% false negative rate for a > 2 cm discrepancy and 72% for a > 10% discrepancy, reliance on these metrics to exclude AKF is flawed. Consequently, this study suggests the use of kidney length differentials as a standalone criterion for the decision-making process needs to be reconsidered.

Echoing our findings, Akoh et al. [6] noted a modest but statistically significant correlation (*r* = 0.333, *P* = 0.005) between renal lengths measured by ultrasound and split renal function determined by MAG3 scans. Their study also uncovered cases where a longer kidney corresponded with reduced function, further complicating the assumption that larger kidney size equates to superior function. These parallel findings reinforce the notion that kidney length, whether assessed by CT or ultrasound, is an unreliable surrogate for functional assessment in prospective kidney donors.

### Kidney and cortical volumes

The assessment of kidney and cortical volumes offers a more promising sensitivity in detecting AKF, with observed rates of 80% for kidney volumes and 88% for cortical volumes when discrepancies exceed 10%. Despite these relatively high sensitivities, the attendant false negative rates—20% for kidney volumes and 12% for cortical volumes—suggest their standalone utility as screening tools remains questionable. These rates imply that a notable minority of potential donors with significant AKF could be erroneously cleared for donation should we rely solely on these CT volumetric measurements.

A recent retrospective cohort study by Montgomery et al found a weak correlation between their split [radionuclide] scan ratio and cortical volume ratio (ρ = 0.361) despite showing a moderate correlation between the cortical volume ratio and the post-donation eGFR [11]. Another retrospective study by Gardan et al also showed a weak to moderate correlation between cortical volume and pre-donation split renal function (*r* = 0.35–0.48) [12].

Due to a range of correlation values yielded from different retrospective studies, a meta-analysis by Habbous et al [5] pooled the Pearson correlation coefficients from 19 studies (*n* = 1479). The meta-analysis suggested a significant correlation between CT-derived split renal volume and radionuclide scan measurements of split renal function (*r* = 0.74, CI 0.61–0.82) and both measurements reliably predicted post-donation eGFR at 12 months (*r* = 0.75 and 0.73 respectively). However, as part of the same paper and in addition to the meta-analysis, Habbous et al performed their own retrospective cohort study of 115 kidney donors and found weak correlations between kidney volume, kidney length and pre-donation split renal function (*r* = 0.22 and 0.24 respectively), which directly contradicts the findings of the meta-analysis section of the same paper [5]. To explain this, the paper is clear that there is very substantial heterogeneity across the included studies (I^2^ = 94%, *p* < 0.0001). This suggests that the correlation achieved between renal volume and split renal function as measured by radionuclide imaging is highly centre-dependent. Furthermore, only 7 studies in the meta-analysis identified a difference in split renal function of > 10%. On analysis of these 7 studies, the pooled positive predictive value of CT-derived renal volume was comparable to our study at 40% as was their negative predictive value of 86%. The sensitivity was 35% and specificity was 88%. Therefore, the meta-analysis concludes that, while a correlation between renal volume and split renal function may exist, this observed correlation is highly centre-dependent. They advocate for future prospective studies to answer the question of whether CT-derived metrics can be reliable and reproducible. The current variability between centres makes CT-derived metrics an unreliable proxy for split renal function. This supports our conclusion that CT volumetry, although informative, cannot singularly determine AKF with the requisite accuracy in screening prospective donors.

These findings highlight our current limited understanding and the need for a cautious approach when considering CT volumetric data in the preoperative evaluation of living kidney donors.

### Split renal function and clinical outcomes

This study attempts to answer the question of whether CT-derived metrics can accurately predict split renal function. It logically follows to then ask if a difference in split renal function of > 10% actually leads to clinically relevant difference in donor outcomes. Our analysis did not collect post-donation eGFR results in donors or recipients and future studies should aim to address the question of whether a difference in split renal function of > 10% has clinical significance. Crucial evidence has already been presented by Seo et al [13] who performed a retrospective cohort study of 217 living kidney transplant cases where the donors underwent radionuclide imaging with both recipients and donors undergoing 12 month follow-up to assess the change in eGFR. Interestingly, there was no association between the recipient’s eGFR (at 12 months post-donation) and whether they received the higher or lesser functioning kidney. However, donors in the study who donated the higher functioning kidney did have poorer renal function at 12 months, despite the fact that no donor had a difference in split renal function of > 10%. This suggests that there are clinical implications for the donor when the better functioning kidney is removed and further studies are required to confirm this finding.

### Significance of study

Our study contributes to the existing literature in two primary ways. Firstly, it joins the other published study where prospective kidney donors are uniformly subjected to CT and functional renal imaging. All other published studies have eligibility criteria for functional imaging, or their study population was less than 30 [6, 14–19]. Our approach mitigates the possible selection bias prevalent in other studies. The comparable study corroborates our findings, demonstrating a similarly weak correlation between kidney volume and function, thus reinforcing the need for a more reliable diagnostic tool [16].

Secondly, we have evaluated the sensitivity and specificity of UK guidelines for pre-donation renal imaging. Our analysis reveals that the current guidelines may not accurately identify candidates who require functional renal imaging. This data helps inform future guideline development to ensure that all prospective donors are assessed for differential renal function, which we suggest is incompletely described by kidney size measurements. The current study provides evidence that split renal function assessment be a standard part of the evaluation for all potential kidney donors to safeguard against inadvertent harm and align donor selection processes with the best evidence available.

### Limitations

A notable limitation is the exclusion of patients who underwent DMSA scanning but not CT imaging. At our centre, both tests are completed for all patients, but this is not the case at other centres. This exclusion could introduce a selection bias, as potential contraindications for donation might preclude the necessity of CT imaging, leading to a study cohort that may not fully represent the broader population of prospective donors. Consequently, our findings might not be generalisable to all individuals undergoing initial screening.

Furthermore, the kidney volume assessments were conducted by a single operator, introducing the possibility of observer bias. However, to mitigate this risk and enhance the reliability of our measurements, a senior radiologist performed a thorough double-read of the initial CT volume measurement protocols during the early phase of data collection.

Lastly, the study’s insights are derived from a single centre within a specific region of the UK and may not capture regional variations in donor characteristics or medical practices. The cohort size, while adequate for initial hypotheses, is relatively small. Future studies with larger, more diverse populations across multiple centres are imperative to validate and possibly extrapolate our findings, ensuring robust, widespread clinical applicability for the detection of AKF in potential kidney donors.

## Conclusions

The findings of this study reveal significant limitations in the use of CT-derived bipolar kidney lengths and volumes as diagnostic indicators of differential kidney function in the evaluation of living kidney donors. The sensitivity and specificity of these measurements are inadequate for reliable identification of clinically significant variations, which raises concerns about their current application in pre-donation assessments. Our data suggests further consideration is required to characterise optimal live donor evaluation and provides evidence to inform future guideline development. Further studies are required to confirm that accurate kidney selection leads to optimal post-donation outcomes for donors and recipients.

### Electronic supplementary material

Below is the link to the electronic supplementary material.

Supplementary Material 1

## Data Availability

Access to anonymised data regarding kidney lengths and volumes is at the discretion of the corresponding author.

## Abbreviations

^99m^Tc-DMSA: Technetium-99m 2,3 dimercaptosuccinic acid
AKF: Asymmetrical Kidney Function
^51^Cr-EDTA: Chromium-51 labeled ethylenediamine tetraacetic acid
CT: Computed Tomography
eGFR: Estimated Glomerular Filtration Rate
ESKD: End-stage kidney disease
KDIGO: Kidney Disease: Improving Global Outcomes
MAG3: Mercaptoacetyltriglycine
mGFR: Measured glomerular filtration rate
