# Supplementary material for: Assessing asymmetrical kidney function in living donors: a retrospective cohort study on CT metrics
Source: BMC Nephrol. 2024 Jul 2;25:214. doi: 10.1186/s12882-024-03634-7 (PMC11221179; doi:10.1186/s12882-024-03634-7)

## Figures, tables, and additional files

**Supplementary figure 1**


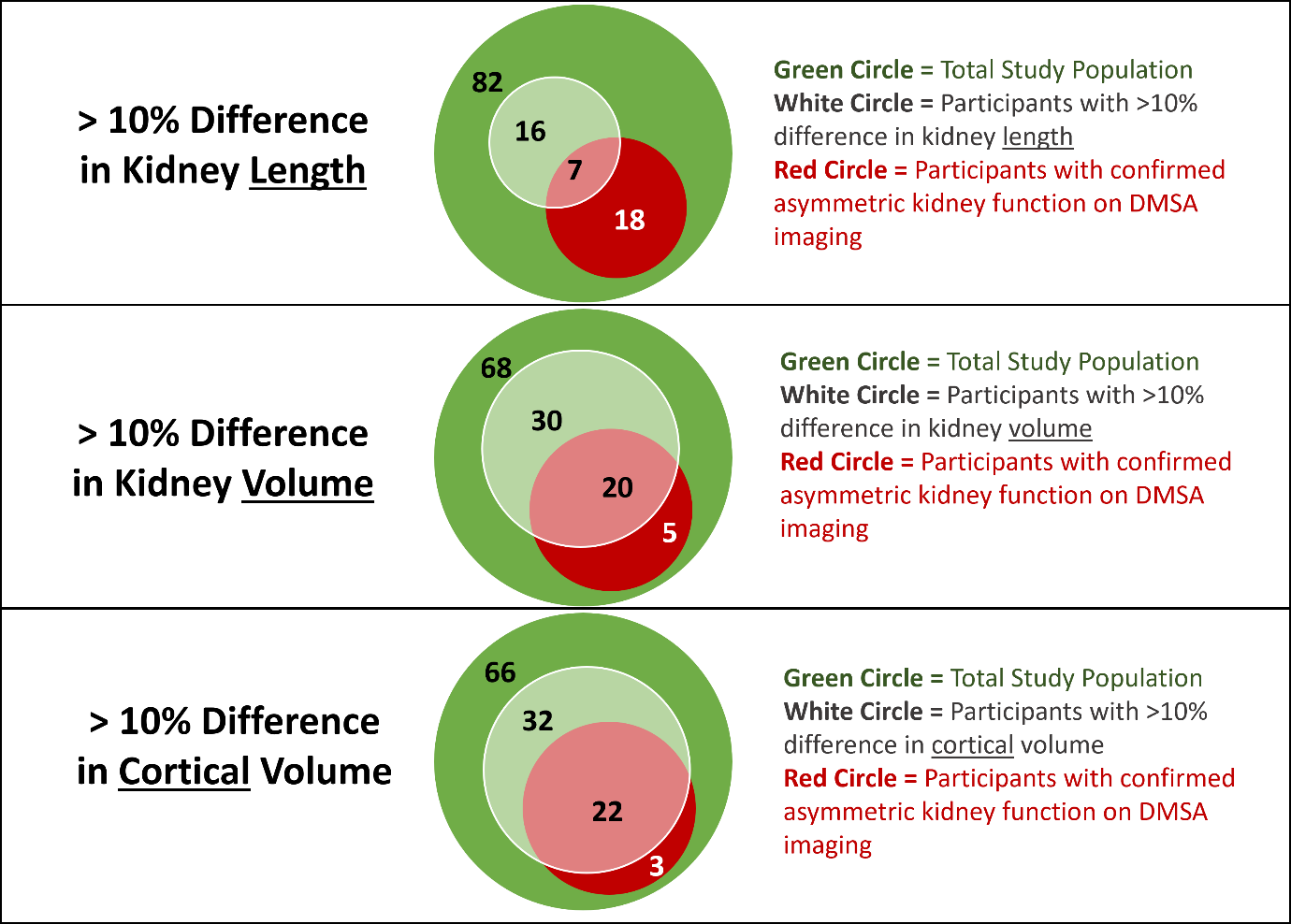


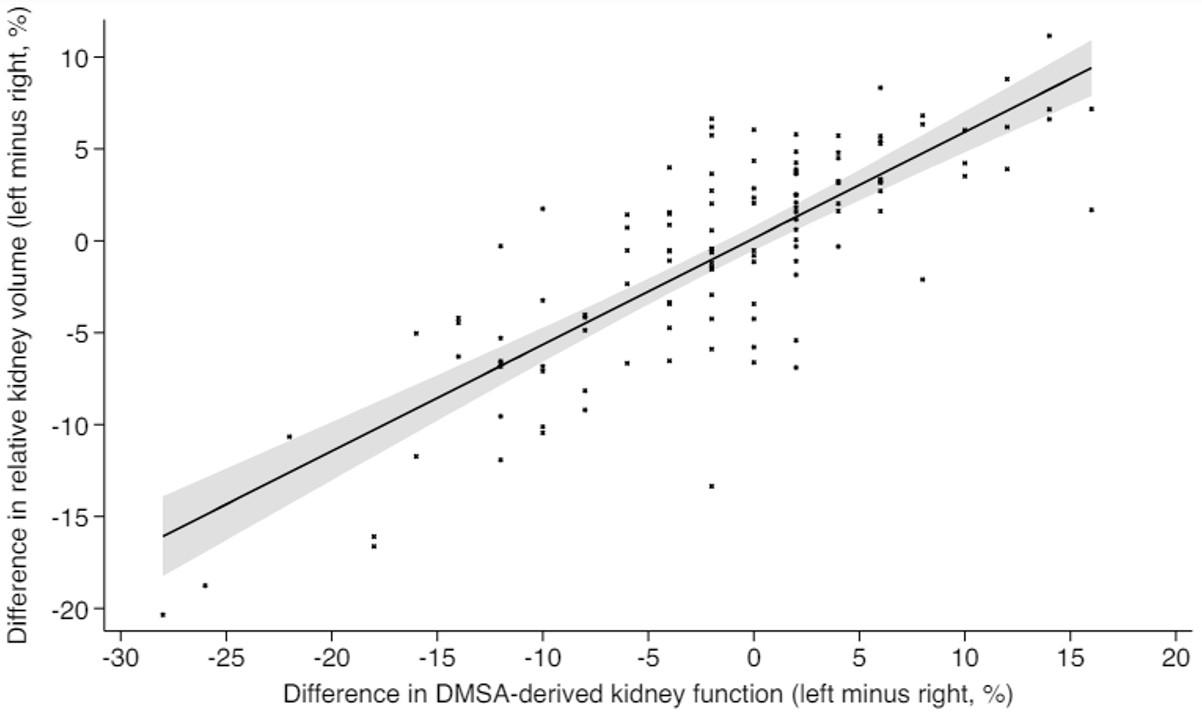
Supplementary figure 2 - Scatter plot of the difference in kidney volume and DMSA-derived differential kidney function. (Regression line and 95% mean confidence interval in shaded area).

Supplementary figure 3 - Scatter plot of the difference in cortical volume and DMSA-derived differential kidney function. (Regression line and 95% mean confidence interval in shaded area).


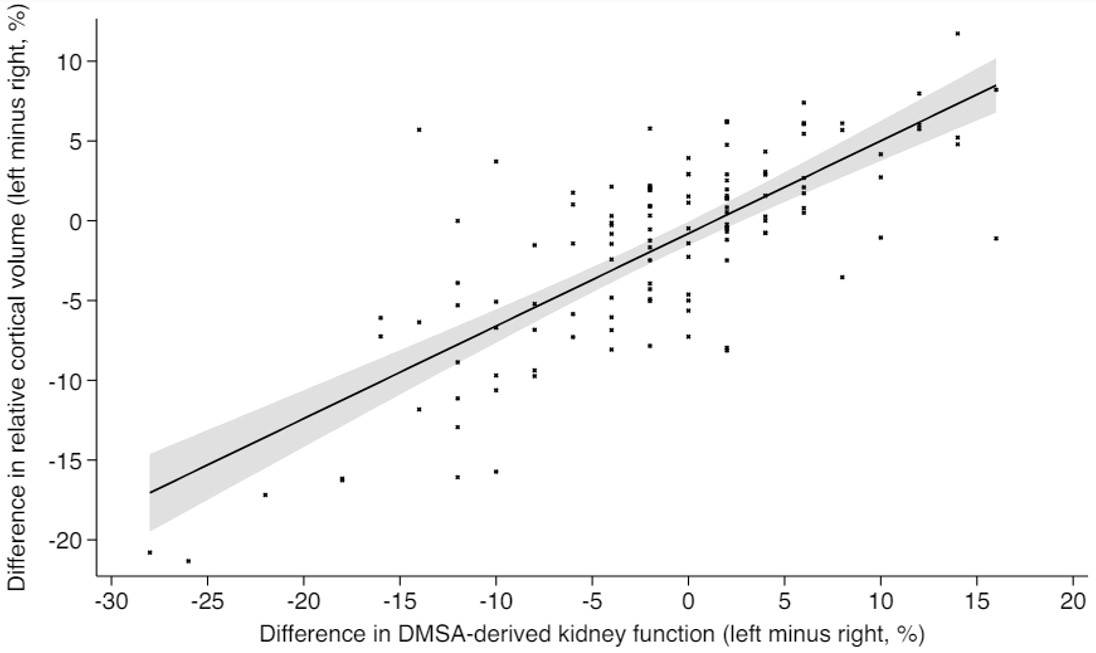

Supplement: Supplementary file 1 — Supplementary Material 1 [file 12882_2024_3634_MOESM1_ESM.docx]
